# Effect of exercise training on ventilatory efficiency in patients with heart disease: a review

**DOI:** 10.1590/1414-431X20165180

**Published:** 2016-06-20

**Authors:** D.M.L. Prado, E.A. Rocco, A.G. Silva, D.F. Rocco, M.T. Pacheco, V. Furlan

**Affiliations:** 1 Grupo TotalCare-Amil, São Paulo, SP, Brasil; 2 Universidade Santa Cecília, Santos, SP, Brasil

**Keywords:** Heart disease, Ventilatory efficiency, Exercise test, Exercise training

## Abstract

The analysis of ventilatory efficiency in cardiopulmonary exercise testing has proven useful for assessing the presence and severity of cardiorespiratory diseases. During exercise, efficient pulmonary gas exchange is characterized by uniform matching of lung ventilation with perfusion. By contrast, mismatching is marked by inefficient pulmonary gas exchange, requiring increased ventilation for a given CO_2_ production. The etiology of increased and inefficient ventilatory response to exercise in heart disease is multifactorial, involving both peripheral and central mechanisms. Exercise training has been recommended as non-pharmacological treatment for patients with different chronic cardiopulmonary diseases. In this respect, previous studies have reported improvements in ventilatory efficiency after aerobic exercise training in patients with heart disease. Against this background, the primary objective of the present review was to discuss the pathophysiological mechanisms involved in abnormal ventilatory response to exercise, with an emphasis on both patients with heart failure syndrome and coronary artery disease. Secondly, special focus was dedicated to the role of aerobic exercise training in improving indices of ventilatory efficiency among these patients, as well as to the underlying mechanisms involved.

## Introduction

Cardiopulmonary exercise testing (CPX) has proven useful for quantifying aerobic capacity and is, therefore, valuable for identifying exercise tolerance in patients with cardiac disease (1–3). Furthermore, CPX responses reflecting ventilatory inefficiency during exercise have attracted a great deal of interest (3–5). Notably, previous investigations (6–9) have shown that patients with heart disease exhibit ventilatory inefficiency, suggesting the existence of mixed impairment of multiple organ systems. In this context, the etiology of increased and inefficient ventilatory response to exercise in cardiac disease is multifactorial, involving both peripheral (e.g., locomotor muscle afferents and oxidative metabolism) and central (e.g., ventilation/perfusion mismatch) mechanisms (6,8,9).

Importantly, aerobic exercise training has been recommended as non-pharmacological treatment for patients with a variety of different comorbidities (10–12). In this respect, a growing body of research (6,7,13,14) has documented increased aerobic fitness in patients with cardiac disease after aerobic exercise training. In addition, previous studies (6,7,14) have demonstrated improvements in ventilatory efficiency after exercise training programs. The primary purpose of this brief review was to elucidate the pathophysiological mechanisms underlying abnormal ventilatory response to exercise, with special emphasis on patients with heart failure syndrome and coronary artery disease. Secondly, special focus was dedicated to the role of aerobic exercise training in improving the indices of ventilatory efficiency among these patients, as well as to the underlying mechanisms involved. More specifically, these indices include ventilation to carbon dioxide production relationship (VE/VCO_2_) and end-tidal CO_2_ pressure (P_ET_CO_2_).

## Determinants of exercise ventilatory response

Exercise hyperpnea is considered to be one of the major remaining challenges to understanding the integrated multi-system responses to exertion. During physical exercise, there is an increase in metabolic rate and consequently in ventilatory demands. Three physical factors determine the ventilatory response to exercise (15): *1*) metabolic CO_2_ production; *2*) mean arterial CO_2_ tension (PaCO_2_) or set point, and *3*) the physiological dead space to tidal volume ratio (VD/VT). Mathematically, ventilatory response during exercise may be explained using the modified alveolar equation (15): VE = 863 × VCO_2_ PaCO_2_ [(1-VD/VT)], where: 863 is the constant correcting for the different standard conditions used to report ventilatory and gas exchange volumes, i.e. body temperature pressure and saturation (BTPS) and standard temperature and pressure dry (STPD), respectively; VCO_2_: pulmonary CO_2_ output; PaCO_2_: arterial PaCO_2_; VD/VT: physiological dead space to tidal volume.

In healthy subjects, the dynamics of ventilatory response to aerobic exercise are closely coupled to pulmonary VCO_2_. In this context, adequate increases in alveolar ventilation requirements are primordial to wash out metabolic CO_2_. In contrast, the rate of pulmonary CO_2_ exchange is further increased at the exercise intensity associated with metabolic acidosis. In other words, additional CO_2_ is produced by the anion bicarbonate (HCO_3_^-^) component of proton (H^+^) buffering at these work rates. This results in a more rapid rate of change in VCO_2_ relative to oxygen consumption (VO_2_) during incremental exercise tests. It is important to recognize that the extra CO_2_ produced under these conditions is a function of the amount of HCO_3_^-^ decrease in the muscle and blood compartments (15). For instance, the excess CO_2_ released at the mouth during ramp exercise exceeding the ventilatory anaerobic threshold (VAT) is thought to derive from the pool of bicarbonate readily available in the blood and in well perfused tissues including the working muscles, with 0.4 L of CO_2_ released for every 1 mmol/L reduction in plasma bicarbonate concentration (16).

PaCO_2_ is normally regulated by respiratory chemoreception (central or peripheral) displaying a bidirectional pattern in which it is increased or remains unchanged during steady-state exercise (17). It has been suggested that peripheral chemoreceptors increase their sensitivity upon exercise; and that respiratory centers change their ‘set-point' in response to oscillations in remaining PaCO_2_ or H^+^ which are phasic with breathing and increase with exercise (17). In this respect, at exercise levels that induce metabolic acidosis (e.g., above VAT), compensatory hyperventilation occurs, lowering the PaCO_2_ required to constrain the fall in arterial pH (15).

Finally, VD/VT ratio reflects pulmonary gas exchange efficiency during exercise. In normal individuals, VD/VT falls from about 0.30-0.40 units at rest to approximately 0.10-0.30 units at peak exercise (18,19). Importantly, although volume of dead space (VD) increases during exercise because of the end inspiratory expansion of conducting airways, the VD increase is small compared to the increase in total tidal volume (VT) and therefore VD/VT decreases (15). Thus, the ventilation needed to wash out a liter of CO_2_ produced during exercise is proportionally reduced (i.e., enhanced gas exchange efficiency).

It is also important to point out the contribution of neural mechanisms of the exercise hyperpnoea to dynamic muscular exercise in humans. In this context, after exercise onset, there is an initial short period in which ventilation increases, despite any changes in both arterial blood gases and acid-base balance (15). More specifically, both central neural feedforward (i.e., descending locomotor command from the motor cortex may directly influence respiratory centers) and peripheral feedback from exercising muscles have been proposed as mediators (15,20). In this sense, Fink et al. (20) demonstrated motor cortical involvement in the control of breathing during exercise in male subjects, through positron emission tomography. In respect to peripheral feedback, group III and IV afferent neurons innervating contracting locomotor muscle are related to increase ventilatory response during rhythmic exercise in humans (21,22). For instance, with the onset of exercise, contraction induced mechanical and chemical stimuli begin to activate receptors on the terminal end of both thinly myelinated (group III, mechanoreflex) and unmyelinated (group IV, metaboreflex) nerve fibers located within skeletal muscle. This activation increases the discharge of these thin afferent muscle fibers, which project via the dorsal horn of the spinal cord to the nucleus of the solitary tract and the medullary cardiorespiratory controller neurons. In fact, the stimulation of limb locomotor groups III-IV afferent fibers (ergoreflex activation) clearly has the capability to increase ventilatory response to rhythmic exercise (21,22). For instance, when group III/IV afferent muscle fibers from the lower limbs are pharmacologically blocked with intrathecal fentanyl during rhythmic leg exercise, circulation and pulmonary ventilation are substantially compromised (23). In this sense, significant hypoventilation is observed in the steady state exercise with pharmacologically blocked afferent muscle fibers (+3 to 8 mmHg mean increase in P_ET_CO_2_) (21).

## Measurement of ventilatory efficiency during exercise

The analysis of ventilatory efficiency during CPX has proven useful for assessing the presence and severity of both heart (9,24–26) and lung (27,28) diseases. During exercise, efficient pulmonary gas exchange is characterized by uniform matching of lung ventilation with perfusion. By contrast, mismatching is marked by inefficient pulmonary gas exchange, requiring increased ventilation for a given CO_2_ production. Interestingly, from a mathematical standpoint VE/VCO_2_ can be described as: {VE/VCO_2_ = k/[(PaCO_2_) × (1-VD/VT)]}.

This implies that factors related to low PaCO_2_ set point and abnormally high dead space fraction (elevated VD/VT) associated with uneven ventilation-perfusion contribute to an excessive ventilatory response to exertion.

Currently, several different methods are used to assess ventilatory efficiency during exercise, such as VE/VCO_2_ relationship and P_ET_CO_2_. With regard to VE/VCO_2,_ there are a number of alternatives to express the VE/VCO_2_ relationship during exercise (29–31), such as: *1*) slope of VE versus VCO_2_ from beginning of exercise to respiratory compensation point (VE/VCO_2_ rest-RCP) or up to peak exercise (VE/VCO_2_ rest-Peak); *2*) VE/VCO_2_ ratio measured at VAT (VE/VCO_2_ @VAT); *3*) VE/VCO_2_ determined by averaging the three lowest consecutive 0.5-min data points (lowest VE/VCO_2_), and *4*) VE/VCO_2_ ratio, measured during submaximal constant workload exercise.

It is important to emphasize that VE/VCO_2_ ratio progressively increase with age and tend to be slightly lower in men than in women (31). In addition, the highest VE/VCO_2_ slope values (e.g., >34 units) contribute to excess ventilation during exercise observed in patients with heart disease. Furthermore, it is not unusual to encounter values >45 in patients with severe heart failure or pulmonary hypertension (5,32).

**Figure 1.**
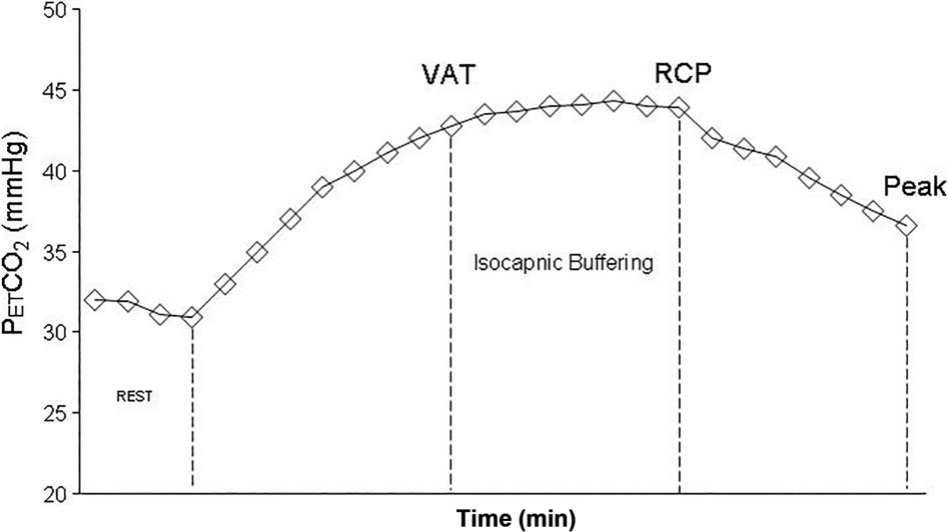
End-tidal CO_2_ pressure (P_ET_CO_2_) response during incremental exercise test in a healthy subject. VAT: ventilatory anaerobic threshold; RCP: respiratory compensation point.

End-tidal PCO_2_ is defined as the highest partial pressure of CO_2_ at the end of expiration during tidal breathing. P_ET_CO_2_ is used as a noninvasive index that is considered a good indicator for evaluating the ventilation/perfusion relationship in patients over a wide range of conditions (33–35). In healthy individuals the normal pattern of P_ET_CO_2_ response during graded exercise test is a progressive increase by approximately 5-8 mmHg above resting values at VAT. Thereafter, P_ET_CO_2_ becomes relatively constant or increases slightly until the onset of the respiratory compensation point during the isocapnic buffering period, and finally decreases progressively until the point of fatigue (Figure 1). The physiological determinants of P_ET_CO_2_ are: *1*) metabolic rate (e.g., the rate of increase in mixed venous PCO_2_); *2*) breathing pattern (e.g., deepness of the previous inspiration (VT) and duration of the exhalation (expiratory time), and *3*) increase in cardiac output and pulmonary blood flow, leading in better perfusion of the alveoli and reduced dead space (18).

With respect to P_ET_CO_2_ analysis during exercise, the following methodologies are used: *1*) change in P_ET_CO_2_ from rest to VAT (Δ P_ET_CO_2_ rest-VAT); *2*) P_ET_CO_2_ measured at VAT, highest value and peak of exercise, and *3*) P_ET_CO_2_ measured during submaximal constant workload exercise (30,33,36).

**Figure 2.**
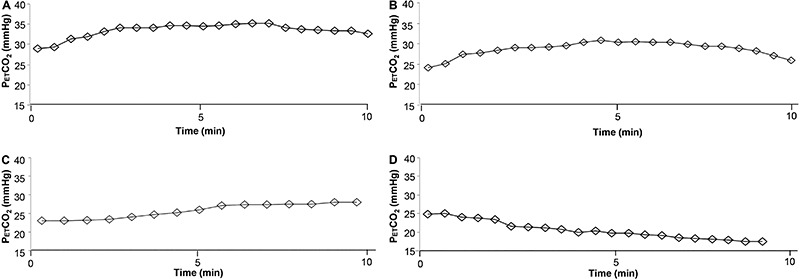
End-tidal CO_2_ pressure (P_ET_CO_2_) response during incremental exercise test in 4 patients with chronic heart failure of progressive severity. *Panel A*, A 69-year-old male patient with dilated cardiomyopathy (left ejection fraction: 60%; peak oxygen consumption (VO_2_): 23.7 mL·kg^-1^·min^-1^; VE/VCO_2_ slope (rest-peak): 33.0). P_ET_CO_2_ at rest was 29.0 mmHg and change in P_ET_CO_2_ from rest to the highest value attained during exercise (ΔP_ET_CO_2_ rest-exercise) was 6.3 mmHg. *Panel B*, A 58-year-old male patient with ischemic heart failure (left ejection fraction: 42%; peak VO_2_: 19.0 mL·kg^-1^·min^-1^; VE/VCO_2_ slope (rest-peak): 40.5). P_ET_CO_2_ at rest was 25.1 mmHg and ΔP_ET_CO_2_ rest-exercise was 5.8 mmHg. *Panel C*, A 60-year-old female patient with ischemic heart failure (left ejection fraction: 38%; peak VO_2_: 13.1 mL·kg^-1^·min^-1^; VE/VCO_2_ slope (rest-peak): 47.1). P_ET_CO_2_ at rest was 23.0 mmHg and ΔP_ET_CO_2_ rest-exercise was 5.0 mmHg. *Panel D*, A 79-year-old female patient with Chagas cardiomyopathy (left ejection fraction: 29%; peak VO_2_: 10.4 mL·kg^-1^·min^-1^; VE/VCO_2_ slope (rest-peak): 63.8). P_ET_CO_2_ at rest was 24.9 mmHg and ΔP_ET_CO_2_ rest-exercise failed to increase.

**Figure 3.**
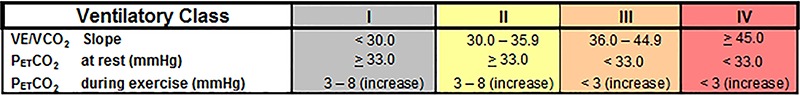
Four progressively worse ventilatory classes. P_ET_CO_2_: end-tidal CO_2_ pressure; VE/VCO_2_: ventilation to carbon dioxide production relationship. Adapted from reference (32).

For instance, a progressive decrease or lower increase in P_ET_CO_2_ seems to be associated with blunted cardiac output response to exercise in cardiovascular disease (35,36) (Figure 2). In addition, it is important to point out that Guazzi et al. (33) proposed a classification of ventilatory inefficiency based on four classes using both VE/VCO_2_ slope and P_ET_CO_2_ parameters (Figure 3).

## Ventilatory inefficiency in heart disease

There is a growing body of evidence indicating impaired ventilatory efficiency in heart disease (6–9,30,37). In this context, a previous study (37) has shown lower ventilatory efficiency in chronic heart failure (CHF) patients when compared to healthy peers (VE/VCO_2_ slope rest-peak=38.9±8.7 *vs* 28.6±3.9 units, respectively). In the same context, Woods et al. (8) performed a submaximal exercise test (65% of peak VO_2_) on a cycloergometer in patients with moderate-severe heart failure and in healthy controls. The authors observed greater VE/VCO_2_ ratio values during submaximal exercise (45±9 *vs* 30±4 units, respectively) and lower P_ET_CO_2_ values (29±6 *vs* 40±3 mmHg, respectively) in patients compared to controls (8).

Notably, substantial evidence (4,5,38–40) demonstrated that ventilatory inefficiency is an independent powerful prognostic marker for cardiac mortality or hospitalization in heart failure patients. A study conducted by Ponikowski et al. (40) with a 49-month follow-up showed that patients with normal peak VO_2_ (≥18 mL·min^-1^·kg^-1^) and VE/VCO_2_ slope ≥34 had a significantly higher mortality rate than patients with normal peak VO_2_ and VE/VCO_2_ slope <34. This investigation was the first to introduce the concept that ventilatory inefficiency during exercise predicts mortality in patients with CHF even when having normal aerobic capacity (40).

Concerning patients with coronary artery disease (CAD), previous investigations have demonstrated lower ventilatory efficiency during exercise, albeit less marked than moderate-severe heart failure patients. In this respect, Eto et al. (41) observed abnormal P_ET_CO_2_ response during exercise in CAD patients. Likewise, Rocco et al. (6) found higher values of VE/VCO_2_ @VAT in CAD patients with reduced aerobic capacity.

## Pathophysiological mechanisms involved in ventilatory inefficiency in heart disease

As outlined previously, inefficient ventilatory response to exercise in heart disease is multifactorial, involving both peripheral (e.g., locomotor afferent muscle fibers and oxidative metabolism) and central (e.g., ventilation/perfusion mismatch) mechanisms. With regard to heart failure syndrome, mechanisms reported for increased ventilatory inefficiency during exercise include: *1*) ventilation-perfusion mismatch; *2*) early occurrence of metabolic acidosis, and *3*) abnormal ergoreflex and chemoreflex control leading to a high ventilatory drive (8,9,42–47).

Previous investigations (44,45,48) found ventilation-perfusion inequalities during exercise in CHF patients. In fact, this finding suggests pathologically high ventilation-perfusion mismatching (i.e., reduced or absent perfusion in well ventilated lung). It should also be highlighted that Wasserman et al. (44) also suggested that structural changes in the lungs and pulmonary vasoconstriction are responsible for the excessive ventilation observed in CHF patients. Additionally, the occurrence of alterations in breathing pattern is associated with a lower rate of increase in VT and higher respiratory rate (e.g., an increase in VD/VT). In this context, patients with heart failure have been shown to develop lung restrictive defects resulting in a shift to low operational lung volumes and a tachypneic breathing pattern (45).

There is also evidence from previous studies (49–51) that CHF patients may demonstrate impaired skeletal muscle metabolism intrinsic to reduced mitochondrial oxidative capacity. Specifically, mitochondrial enzymes (citrate synthase, and succinic dehydrogenase) and enzymes involved in β-oxidation have been shown to be decreased (51,52). Notably, a previous study showed a close relationship between a decrease in cytochrome oxidase activity and reduction in peak VO_2_ in heart failure patients (51). Additionally, skeletal muscle characteristics have important roles on substrate and oxygen utilization during physical exercise. Patients with heart failure have shown skeletal muscle abnormalities related to decreases in oxidative type I fibers and increases in glycolytic type IIb fibers (49,52). Together, these alterations indicate a shift from aerobic to anaerobic metabolism, which results in early occurrence of metabolic acidosis and exercise intolerance.

Furthermore, the blunted cardiac output response during exercise observed in CHF patients is associated with a decrease in convective O_2_ transport to working muscle, thereby increasing anaerobic glycolysis (9). Thus, enhanced lactic acidosis at lower work rates leads to an increase in H^+^ stimulus, augmenting ventilation drive and fatigue in these patients.

It should also be noted that afferent feedback linked to receptors in locomotor muscle (ergoreflex activation) originating from groups III and IV (myelinated and unmyelinated, respectively) contributes to altered ventilatory control during exercise in heart failure patients (42,43). More specifically, afferent neural feedback from skeletal muscle is an established regulator of cardiovascular and respiratory control during exercise in both animal and healthy humans (43). For instance, ergoreflex activation during exercise is stimulated by mechanical distortion of the receptive field (group III) and by products of metabolism (group IV). An interesting study carried out by Olson et al. (43) found that inhibiting afferent feedback from locomotor muscle via intrathecal opioid administration significantly reduced ventilatory response during exercise in CHF patients when compared to placebo (VE/VCO_2_ ratio = 33.8±1.1 *vs* 28.1±0.7 units, respectively).

Further pathophysiological evidence for increased ventilatory inefficiency response to exercise in heart failure can be found in impaired chemoreflex control (9,46). Chemoreflexes are the dominant control mechanisms regulating ventilatory responses to changes in arterial oxygen and CO_2_ content. Narkiewicz et al. (46) showed that CHF patients are characterized by greater activation of ventilatory responses to central chemoreceptor activation by hypercapnia (P_ET_CO_2_=50 mmHg) than normal subjects. Moreover, Giannoni et al. (47) demonstrated an enhanced chemosensitivity to hypoxia in systolic heart failure patients. Noteworthy, in the same investigation the authors observed that an increase in both hypoxic and hypercapnic chemosensitivity is related to autonomic imbalance, neurohormonal activation and ventilatory inefficiency in CHF patients.

**Figure 4.**
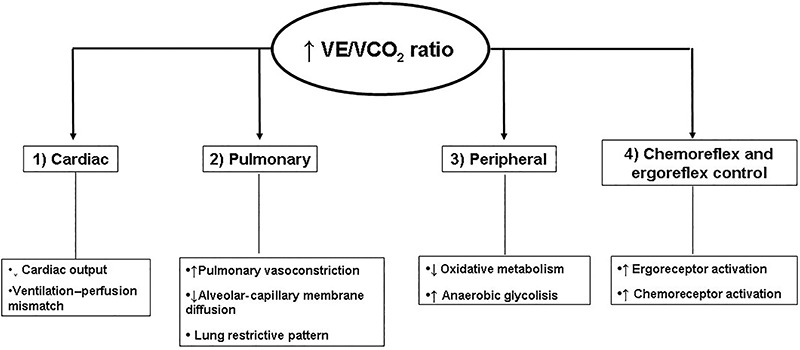
Potential mechanisms suggested for reduced ventilatory efficiency (increased ventilation to carbon dioxide production relationship (VE/VCO_2_) in cardiac disease.

This evidence, taken together with the aforementioned findings, suggests that both peripheral and central mechanisms may be associated with reduced ventilatory efficiency in heart failure patients (Figure 4).

Regarding CAD patients without CHF, it seems unlikely that these individuals have alterations in breathing pattern during exercise. However, the ventilatory inefficiency in CAD patients may be related with reduced aerobic capacity (6) and high ventilation-perfusion mismatching (41). In this context, Eto et al. (41) showed an association between impaired cardiac inotropic function and ventilatory inefficiency in patients after acute myocardial infarction.

Moreover, a previous study showed that alterations in chemoreflex control, possibly through excessive sympathetic tone, may also be associated (50). Specifically, the enhanced central hypercapnic chemosensitivity increases sympathetic outflow and therefore an increase in ventilatory response is observed (45). In this context, Tomita et al. (53) demonstrated that CAD patients with ventilatory inefficiency (VE/VCO_2_ slope=35±5) had increased CO_2_ chemosensitivity. Further investigations are necessary to confirm these issues.

## Effect of exercise training on ventilatory inefficiency in heart disease

Substantial evidence (6,7,10,13,14,41,48,50,53) supports the benefits of exercise training-based cardiac rehabilitation in heart disease. In fact, previous investigation have shown that when patients with heart disease are submitted to dynamic exercise training programs, aerobic capacity can improve by up to 25% (54). Additionally, a growing body of research (6,7,10,13,14,41,48,53) shows improved ventilatory efficiency in heart patients after exercise training. Furthermore, a VE/VCO_2_ slope reduction of between 6 and 23% has been observed in chronic heart failure patients after exercise training programs. In this respect, Gademan et al. (14) showed improvement in ventilatory efficiency (VE/VCO_2_ slope, pre-=35.8±3.9 *vs* post-training=31.0±6.1 units, Δ change=-14%) in CHF patients after exercise training. Similarly, Meyer et al. (55) observed a 14.6% decrease in VE/VCO_2_ slope after short-term exercise training (3 weeks) in patients with severe CHF.

**Figure 5.**
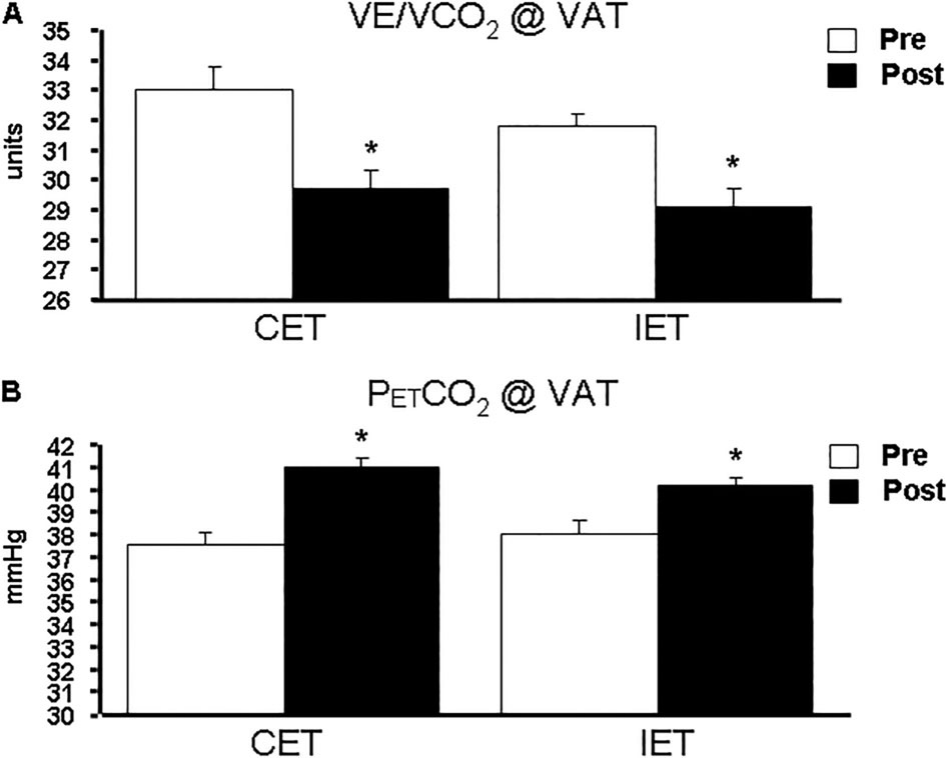
Effects of continuous exercise (CET) and interval exercise training (IET) on indices of ventilatory efficiency in coronary artery disease patients. *Panel A*, Ventilation to carbon dioxide production relationship (VE/VCO_2_) at ventilatory anaerobic threshold (VAT); *Panel B*, End-tidal CO_2_ pressure (P_ET_CO_2_) at VAT. Pre: pre-intervention; Post: post-intervention. *P<0.05 *vs* pre-intervention. Adapted from reference (6).

In CAD patients, Tomita et al. (53) found a decrease in VE/VCO_2_ slope after aerobic exercise training. Furthermore, Rocco et al. (6) evaluated the effects of continuous and interval exercise training on P_ET_CO_2_ response during graded exercise tests. Interestingly, after a 12-week exercise training program, CAD patients submitted to both exercise training methods showed a similar response in decrease ventilatory inefficiency (6) (Figure 5)

**Figure 6.**
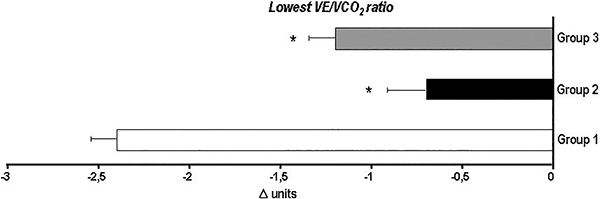
Absolute changes in ventilatory efficiency among coronary artery disease patients after aerobic exercise training program. Group 1 (n=34, peak VO_2_ <17.5 mL·kg^-1^·min^-1^; lowest VE/VCO_2_ ratio 33.5); group 2 (n=67, peak VO_2_ >17.5 and <24.5 mL·kg^-1^·min^-1^; lowest VE/VCO_2_ ratio 29.7) and group 3 (n=22, peak VO_2_ >24.5 mL·kg^-1^·min^-1^; lowest VE/VCO_2_ ratio 29.0). Note that group 1 showed a greater decrease in the lowest VE/VCO_2_ ratio compared to the other two groups. VE/VCO_2_: ventilation to carbon dioxide production relationship; VO_2_: oxygen consumption. *P<0.05 *vs* group 1 (one-way ANOVA). Adapted from reference (7).

In addition, Prado et al. (7) investigated whether aerobic fitness status was associated with greater improvements in ventilatory efficiency in CAD patients after aerobic exercise training. More specifically, 123 patients with CAD were divided into 3 groups: group 1 (n=34, peak VO_2_ <17.5 mL·kg^-1^·min^-1^); group 2 (n=67, peak VO_2_ >17.5 and <24.5 mL·kg^-1^·min^-1^) and group 3 (n=22, peak VO_2_ > 22.5 mL·kg^-1^·min^-1^). After 12 weeks of an aerobic exercise training program, the CAD patients with lower initial aerobic fitness exhibited greater improvements in ventilatory efficiency than the other groups (Figure 6).

Concerning the physiological mechanisms responsible for increasing ventilatory efficiency after exercise training programs, these may be dependent on peripheral and central mechanisms. A determinant role of locomotor muscle abnormalities in limited exercise performance in heart failure is well documented (9,42,43,49,50). Notably, aerobic exercise training not only improves peak VO_2_ but also promotes a number of significant changes in the exercising limbs, such as increased O_2_ uptake and arteriovenous O_2_ difference, as well as decreased lactate accumulation and ergoreflex activation (50). Supporting this notion, Van Laethem et al. (56) attributed improvements in muscle receptor reflexes and skeletal muscle metabolism after aerobic exercise training to more efficient ventilation during exercise in CHF patients. Moreover, a previous study (42) demonstrated that, after exercise training, an improvement in ergoreflex control was associated with a 46.9% decrease in ventilatory response during exercise in CHF patients. Furthermore, Adamopoulos et al. (50), studying patients with heart failure after 8 weeks of exercise training, found lower phosphocreatine depletion and a smaller increase in adenosine diphosphate at matched submaximal workloads. These findings suggest an improvement in oxidative metabolism related to an increase in mitochondrial content or activity.

With respect to central mechanisms, previous studies (10,57) have demonstrated the beneficial effects of exercise training in improving cardiac output among CHF patients. Coats et al. (10) for example, in 17 patients with moderate to severe CHF that underwent 8 weeks of exercise training, observed an increase in submaximal cardiac output (pre-=5.9 *vs* post-training=6.7 L/min, respectively) and peak cardiac output (pre-=6.3 *vs* post-training=7.1 L/min, respectively). Based on these findings, it seems reasonable to speculate that an increase in cardiac output after exercise training programs can improve convective O_2_ transport to the working locomotor muscle, thus reducing both metabolic disturbance and ergoreflex activation during exercise in CHF patients. In fact, limited cardiac output increase during exercise and oxidative metabolism impairment in working locomotor muscle are associated with the development of early lactic acidosis during exercise in heart failure patients.

Importantly, Guazzi et al. (48) showed that exercise training significantly improved alveolar-capillary membrane capacity (D_M_) in CHF (D_M_, pre-=31.4±3.2 *vs* post-training=36.1±3.8 mL·min^-1^·mmHg^-1^, respectively). According to the authors, this finding can be explained by higher cardiac output and better diffusion-perfusion matching (i.e., improved ventilation/perfusion ratio). This result shows that impairment in lung diffusion among CHF patients is involved in ventilatory inefficiency and that there is a link between changes in lung function and improvement in exercise capacity with exercise training.

**Figure 7.**
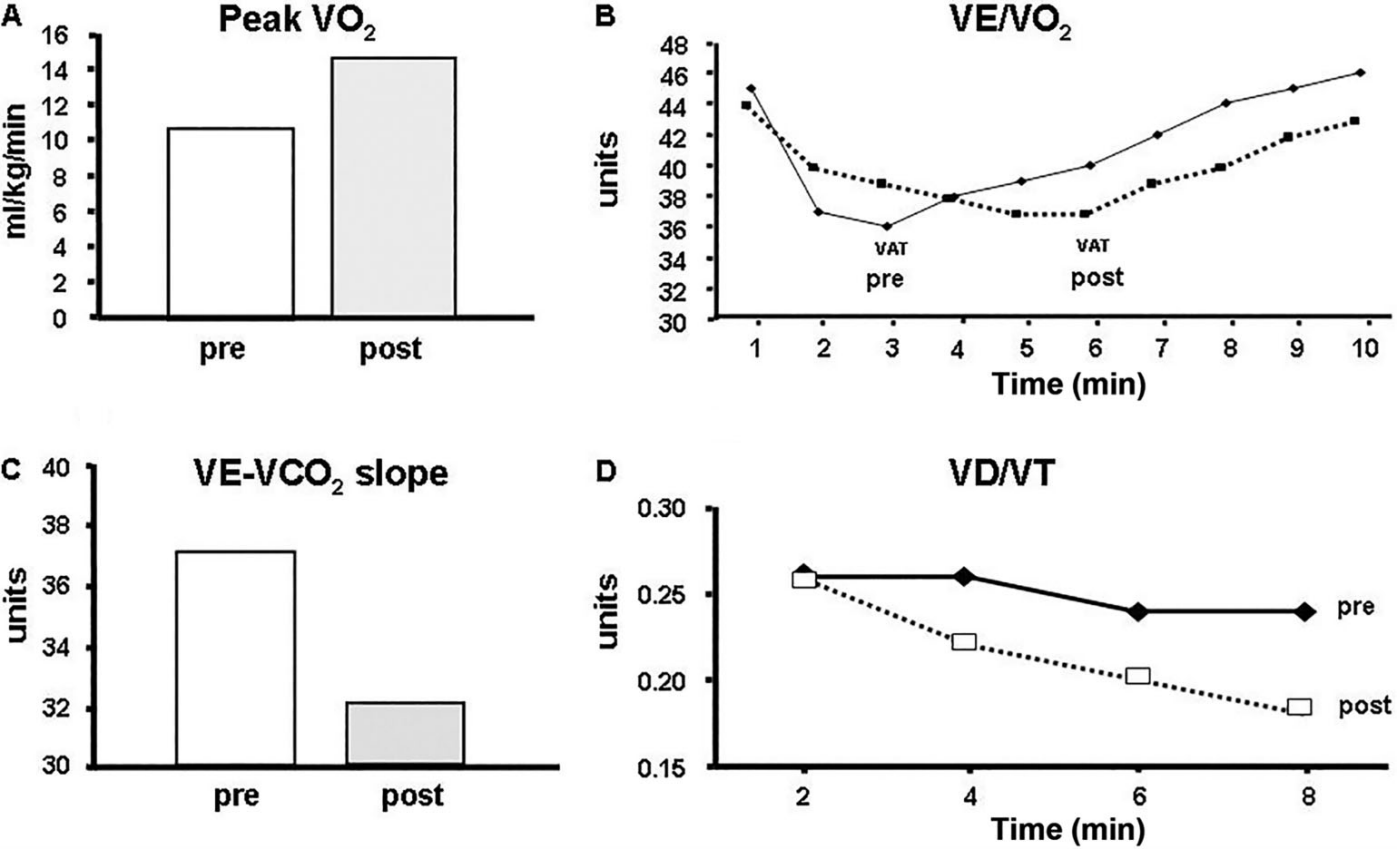
Effect of a 12-week aerobic exercise training program in an 82-year-old female patient with coronary artery disease. *Panel A*, peak oxygen consumption (VO_2_); *panel B*, VE/VO_2_, ventilatory equivalent for oxygen; *panel C*, ventilation to carbon dioxide production relationship (VE-VCO_2_) slope (rest-peak); *panel D,* dead space to tidal volume ratio (VD/VT). Note that after aerobic exercise training the patient showed improvement in both aerobic fitness and ventilatory efficiency (*panels A* and *C*, respectively). The patient demonstrated a marked shift to the right of the VAT suggesting improvement in aerobic efficiency (*panel B*). In addition, the patient demonstrated a sharper reduction in VD/VT showing improvement in gas exchange efficiency (*panel D*). pre: pre-intervention; post: post-intervention; VAT: ventilatory anaerobic threshold; VD/VT: estimate physiological dead space to tidal volume ratio.

Notably, previous studies support the notion that chemoreflex abnormalities play a role in ventilatory inefficiency in heart failure (46,47). In this context, CHF patients have enhanced chemoreflex sensitivity through augmented afferent input from the carotid body (47). Importantly, the beneficial effects of exercise training on peripheral chemoreflex sensitivity in heart failure patients have also been documented (58). For instance, improvement in peripheral chemoreflex control was normalized after exercise training in a rabbit model of heart failure (58). These findings are associated to reversal of alterations in angiotensin II systems and nitric oxide expression in the carotid body [59]. With respect to central CO_2_ chemosensitivity, to date, little is known about the effects of exercise training on hypercapnic chemoreflex sensitivity in CHF patients. However, concerning CAD patients, a previous study (53) showed that reduced VE/VCO_2_ slope after exercise training was correlated with attenuation in CO_2_ chemosensitivity. Interestingly, the suppression of hypercapnic chemoreflex response was associated with improvement in the sensation of dyspnea during exercise in CAD patients. Additionally, other investigations suggest that both oxidative metabolism and ventilation/perfusion matching are factors contributing to reduced ventilatory inefficiency after aerobic exercise training in CAD patients (6,7,53) (Figure 7).

For example, Rocco et al. (6) showed a positive association between P_ET_CO_2_ and VO_2_ at VAT, suggesting that increases in P_ET_CO_2_ after aerobic exercise training were related to improvements in aerobic metabolism. In the same study, the authors also observed that higher P_ET_CO_2_ values after exercise training were associated with lower VD/VT at VAT. Furthermore, Prado et al. (7) demonstrated after 12 weeks of aerobic exercise training, that increased peak O_2_ pulse (pre- 10.1±0.6 *vs* post-training 16.1±3.1 mL/bpm, respectively) was associated with a decrease in the lowest VE/VCO_2_ ratio among CAD patients with lower aerobic fitness. Overall, the aforementioned findings suggest an improvement in ventilation/perfusion matching during exercise.

## Conclusion

Exercise limitation is an important manifestation accompanied by ventilatory inefficiency in patients with cardiorespiratory disease. Analysis of ventilatory efficiency during exercise has proven useful for assessing the presence and severity of heart disease even in patients without apparent exercise limitation. The bulk of evidence has shown that improvements in ventilatory efficiency among heart patients after exercise training may be dependent on peripheral and central mechanisms. Taken together, these findings suggest that exercise programs have clinically significant effects because the low ventilatory efficiency observed in patients with heart diseases constitutes an important predictor of cardiovascular mortality.
